# Corrigendum

**DOI:** 10.1002/ehf2.12444

**Published:** 2019-05-07

In the paper by Zabel et al.,^1^ the following correction should be noted:

On page 8, second column, paragraph 4 on the Polish centers, MUL CKD Hospital in Lodz/Poland was credited with “I. Cygankiewicz, P. Ptaszyński”. Instead it should have been: “I. Cygankiewicz, P. Ptaszyński, K. Kaczmarek, I. Poddebska”.

## References

[1] Zabel M , Sticherling C , Willems R , Lubinski A , Bauer A , Bergau L , Braunschweig F , Brugada J , Brusich S , Conen D , Cygankiewicz I , Flevari P , Taborsky M , Hansen J , Hasenfuß G , Hatala R , Huikuri HV , Iovev S , Kääb S , Kaliska G , Kasprzak JD , Lüthje L , Malik M , Novotny T , Pavlović N , Schmidt G , Shalganov T , Sritharan R , Schlögl S , Szavits Nossan J , Traykov V , Tuinenburg AE , Velchev V , Vos MA , Willich SN , Friede T , Svendsen JH , Merkely B , for the EU‐CERT‐ICD Study Investigators . Rationale and design of the EU‐CERT‐ICD prospective study: comparative effectiveness of prophylactic ICD implantation. ESC Heart Fail 2019; 6: 182–193. 10.1002/ehf2.12367. 30299600 PMC6351896

